# Telemedicine in pediatric rheumatology: this is the time for the community to embrace a new way of clinical practice

**DOI:** 10.1186/s12969-020-00476-z

**Published:** 2020-10-31

**Authors:** Susan Shenoi, Kristen Hayward, Megan L. Curran, Elizabeth Kessler, Jay J. Mehta, Meredith P. Riebschleger, Helen E. Foster

**Affiliations:** 1 Department of Pediatrics, Division of Rheumatology, Seattle Children’s Hospital and Research Center, University of WA, MA.7.110 4800 Sand Point Way NE, Seattle, WA 98105 USA; 2 Department of Pediatrics, Section of Rheumatology, University of Colorado, 13123 E. 16th Ave, B311, Aurora, CO 80045 USA; 3 Department of Pediatrics, Section of Rheumatology, Helen DeVos Children’s Hospital, Grand Rapids, MI. 35 Michigan St NE, Grand Rapids, MI 49503 USA; 4 Division of Rheumatology, Children’s Hospital of Philadelphia, 3501 Civic Center Blvd, Philadelphia CTRB 10109, Philadelphia, PA 19104 USA; 5 Department of Pediatrics, Division of Rheumatology, University of Michigan, 1500 E. Medical Center Dr, Ann Arbor, MI 48109 USA; 6 Professor Pediatric Rheumatology, Population Health Sciences Institute, Newcastle University, The Medical School, Framlington Place, Newcastle upon Tyne, NE2 4HH UK

**Keywords:** Telemedicine, E-visits, Models of care, V-pGALS, Covid-19

## Abstract

**Background:**

The use of telemedicine in pediatric rheumatology has been historically low. The current COVID 19 global pandemic has forced a paradigm shift with many centers rapidly adopting virtual visits to conduct care resulting in rapid expansion of use of telemedicine amongst practices.

**Body:**

This commentary discusses practical tips for physicians including guidance around administrative and governance issues, preparation for telemedicine, involving the multidisciplinary care team, and teaching considerations. We also outline a standard proforma and smart phrases for the electronic health record. A proposed variation of the validated pediatric gait arms legs spine examination (pGALS) called the video pGALS (VpGALS) as a means of conducting virtual pediatric rheumatology physical examination is presented.

**Conclusion:**

This commentary provides a starting framework for telemedicine use in pediatric rheumatology and further work on validation and acceptability is needed.

## Background

The COVID-19 pandemic has forced rapid changes in the way that medical care is delivered worldwide. Virtual care models with remote clinics and video visits (e-visits or telemedicine) have become widespread practice overnight. The adoption of telemedicine in pediatric rheumatology has been limited historically [1] and the importance of physical examination cited as a barrier [2]. Furthermore, regulatory complexity, decreased reimbursement rates and technical limitations have hampered robust development of telemedicine. However, for many providers and families the pandemic has resulted in a need for pragmatism often in the absence of formal training or sophisticated technical support. The unique need to balance social distancing with providing ongoing care to an often immunosuppressed patient population combined with relaxation of regulatory demands has enabled rapid expansion of video visits. The American College of Rheumatology position statement on telemedicine also reflects this rapidly evolving need (https://www.rheumatology.org/Portals/0/Files/Telemedicine-Position-Statement.pdf). This commentary describes practical creative approaches based on our experiences and discusses the potential for telemedicine to address unmet needs in the wider context of pediatric rheumatology.

### Administrative and governance considerations

Guidance for telemedicine clinics is available (Table 1) albeit governance considerations differ between countries and institutions may also have specific requirements. In both the United States (USA) and European Union (EU), *credentialing* of providers to provide telemedicine is entrusted to the *remote site* where they are physically located [3, 4] and providers must be licensed to practice at the remote site and the *originating site* (where the patient is located) [3]. Consent is important and specific concerns relate to the lack of a ‘hands on’ in-person physical examination and more universal concerns regarding privacy and technology issues. *Patient privacy and confidentiality* is paramount and needs to be addressed in the technological requirements with access (at both originating site and remote sites) restricted to individuals essential to facilitate patient care. This may include an in person telepresenter, multiple virtual support staff to help coordinate after visit care, and, other specialists and members of the multidisciplinary team (MDT). Many electronic health record (EHR) systems have compliant telehealth modules (e.g. Health Insurance Portability and Accountability Act (USA) and General Data Protection Regulation (EU). For circumstances in which an EHR module is not available, several commercial vendors (e.g. Zoom, Telehealth) offer ‘standalone’ privacy compliant video platforms. Issues related to *data ownership and security* vary in different regions of the world, depending upon whether telemedicine is considered to be a healthcare service or an information service. It is important to review the contract for services, as different vendors may also have different policies. Both standalone telemedicine platforms and EHR modules offer the ability to capture still images (photographs) or have the capacity for asynchronous communication. The billing systems used for in-person visits are also used for telemedicine visits. Limitations in the observed physical examination make time-based billing more rewarding in many circumstances, but the complexity of pediatric rheumatology medical decision make it a viable alternative in our specialty.

**Table 1:** Resources for Telerheumatology

Telemedicine practice guidance	https://www.americantelemed.org/, https://www.ama-assn.org/practice-management/digital/ama-quick-guide-telemedicine-practice
Practical tips: ● administration ● technical platforms and equipment ● involving the wider clinical team ● preparation of the family ● suggested Dot Phrases for electronic health record and exemplar consent ● suggested examination schedule ● recording proforma ● teaching	http://www.pmmonline.org/doctor/approach-to-clinical-assessment/examination/telehealth
V-pGALS	http://www.pmmonline.org/doctor/approach-to-clinical-assessment/examination/v-pgals

### Practical considerations

Preparation is essential and includes provider related tasks, incorporating the broader MDT and ensuring that family are supported with the requisite equipment and information. We provide resources and practical tips in Table 1.

Patient selection for video visits will be influenced by individual site and situational factors. Generally speaking most established patients can be seen by video visits at least on an intermittent basis. Young patients (< 3 years) are more challenging to keep on task with a virtual joint exam, but a care-giver only visit to discuss symptoms or medication side effects is often feasible. Guidance for consideration of urgent in-person evaluations during the ongoing pandemic is available (https://www.rheumatology.org/Portals/0/Files/Guiding-Principles-Urgent-vs-Non-Urgent-Services.pdf) and include new patient evaluations when the consulting or referring provider indicates urgency, acute flare or ongoing disease activity of a known disease.

The Virtual Exam Section requires creativity depending on the location of the family/patient, consideration of the exam sequence, technical issues and how to cue the family and child to gain optimal views (Table 1). The pGALS assessment is a validated simple basic musculoskeletal (MSK) examination [5] and a proposed variation, called Video-pGALS (V-pGALS – Table 1) includes maneuvers from pediatric regional examination of the musculoskeletal system (pREMS) [6] and suggestions from the authors. V-pGALS offers a structured approach to a cursory overview MSK exam with a focus on range of movement and symmetry. Utilizing the parent or caregiver to feel the joint in question for obvious warmth/swelling or to palpate for point of maximal tenderness can add important information. Our experience suggests that for patients with myositis or muscle complaints, the Childhood Myositis Assessment Score (CMAS) [7] is fairly easy to administer across the virtual platform.

The Actual Visit**.** Effective video visits require adequate clinical staff and administrative support. A call ahead of the scheduled visit helps to prepare the family regarding technology needs and to gather information (e.g. medications, allergies and recent weight). At the start of the visit, obtaining consent is important and a brief scaffolding statement helps to ease anxiety and set expectations. Documentation of discussions with the family and mechanisms to ensure follow up tasks are coordinated and carried through is critical. We provide suggested EHR Dot Phrases and a recording proforma (Table 1).

Multidisciplinary clinics. Some platforms have capability to invite multiple providers and other staff in the virtual visit room. This is useful to have individuals who may be in a different location on the visit, from a different specialty or members of the MDT (e.g. nurse, physical therapist) or other key persons (e.g., social worker, interpreter, or psychologist).

Teaching opportunities. Telehealth clinics provide novel educational opportunities (Practical Tips - Table 1). In the virtual visit, both the trainee and attending can be present simultaneously for the entirety of the visit providing bi-directional opportunity for direct observation and coaching for the trainee (history taking, physical exam, communication skills) and trainee observation of key points that the attending gathers or style of attending counselling. Published tools for assessment that work well in this context are available [8, 9]. The typical precepting model can be reproduced in the virtual setting by temporarily placing the patient and family in the virtual waiting room while the trainee and attending confer and then having the family rejoin the virtual visit with both providers in attendance. Alternatively, for many patients, the trainee can review their assessment and plan with the attending within the video visit while the family is present as a method to confirm the information gathered and demonstrate transparency in the precepting process. This option also allows for the benefit of capturing attending time spent evaluating the patient and this may be important if using time-based billing as only attending physician time can be counted.

### Conclusion and future aspects

The COVID-19 pandemic has provided an opportunity to expand telerheumatology and address workforce challenges around the world [10, 11]. Telemedicine in rheumatology has advantages but the limitations (Table 2) need to be addressed to enable adoption into routine clinical practice. There is need to evaluate the validity and acceptability of the overall quality of care, family and provider experience as well as virtual exam techniques such as V-pGALS and CMAS. There are inherent costs of technology and provider training but once set up, the model may well be cost effective. There is huge potential for networking, education and training, especially in areas of the world with no local specialist provision. The pandemic has undoubtedly brought much health and economic distress to the world and has necessitated pragmatic solutions to clinical care. Such experience has focused minds and provided opportunity for collaboration between providers, clinicians, and families to develop a model of care utilizing technology to complement traditional health care delivery and improve access to care to more children.

**Table 2:** Advantages and Limitations of Telerheumatology

**Advantages**	
Increased access to specialist opinion for families living in rural/remote areas [12] and ability for MDT members to join from different locations in same visit	
Reduced travel time (caregivers and physicians) Reduced missed work (caregivers) [13],	
Reduced missed school (patients), Cost savings (families) [1]	
Video visits can be efficient especially when the provider links directly to the patient	
Some EHR systems provide ability for families to complete questionnaires beforehand	
Education and training. Trainees, including residents, fellows, allied health and medical student teaching could be incorporated to improve exposure to pediatric rheumatology at training centres where this expertise in not available.	
Potential for outpatient or inpatient e-consults to remote hospitals where pediatric rheumatologists are not on staff	
**Limitations**	
Subtle exam findings can be missed. A potential solution is the use of a trained telepresenter (e.g. family doctor, pediatrician, physiotherapist or nurse) or training parents to facilitate the examination.	
Families express preference for in-person visits, even when travel is inconvenient [2]	
Challenges in developing rapport especially with new patients	
Complex or medically serious visits need in person assessment [14]	
Participation in research studies which historically have required in person evaluations (might require creative solutions)	
Shortfalls in network, hardware and software capabilities, either on the provider or patient end can cause inability/ difficulty with connecting, or poor video resolution	
Equipment and training of providers is often costly and time-consuming, with decreased provider acceptance [15].	
Equity issues: Limited access for some families with poor or no internet access or limited data plans, low bandwidth capacity, limited language proficiency, health literacy and technological literacy [15]	
Lack of or inadequate insurance coverage Geographic boundaries may be bound to different telehealth rules based on government and hospital restrictions	
Internet and software platforms may not have security to ensure privacy of video or healthcare data [15].	

## Data Availability

Not applicable.

## Abbreviations

EHR: Electronic Health Record
USA: United States of America
EU: European Union
MDT: Multidisciplinary team
ACR: American College of Rheumatology
PMM: Pediatric Musculoskeletal Matters
MSK: Musculoskeletal
pGALS: Pediatric Gait Arms Legs Spine
V-pGALS: Video –Pediatric Gait Arms Legs Spine
pREMS: Pediatric regional examination of the musculoskeletal system
CMAS: Childhood Myositis Assessment Score
